# *N*-Acyl Homoserine Lactones in Diverse *Pectobacterium* and *Dickeya* Plant Pathogens: Diversity, Abundance, and Involvement in Virulence

**DOI:** 10.3390/s120303484

**Published:** 2012-03-12

**Authors:** Alexandre Crépin, Amélie Beury-Cirou, Corinne Barbey, Christine Farmer, Valérie Hélias, Jean-François Burini, Denis Faure, Xavier Latour

**Affiliations:** 1 Laboratoire de Microbiologie Signaux et Microenvironnement, Normandie Université, Université de Rouen EA 4312, IUT Evreux, 55 rue Saint-Germain, Evreux F-27000, France; E-Mails: alexandre.crepin@etu.univ-rouen.fr (A.C.); corinne.barbey@univ-rouen.fr (C.B.); christine.farmer1@univ-rouen.fr (C.F.); jean-françois.burini@univ-rouen.fr (J.-F.B.); 2 SIPRE—Comité Nord, Station de recherche et de création variétale, Bretteville du Grand Caux F-76110, France; E-Mail: amelie.cirou@isv.cnrs-gif.fr; 3 Institut des Sciences du Végétal, CNRS UPR2355, Avenue de la Terrasse, Gif-sur-Yvette 91198, France; E-Mail: faure@isv.cnrs-gif.fr; 4 FN3PT, Fédération Nationale des Producteurs de Plants de Pomme de Terre, 43-45 rue de Naples, Paris F-75008, France; E-Mail: valerie.helias@rennes.inra.fr; 5 INRA, UMR 1349 IGEPP, Le Rheu Cedex F-35653, France

**Keywords:** soft-rot bacteria, *Solanum tuberosum* L., *N*-acyl homoserine lactones, quorum sensing, AttM lactonase, quorum quenching

## Abstract

Soft-rot bacteria *Pectobacterium* and *Dickeya* use *N*-acyl homoserine lactones (NAHSLs) as diffusible signals for coordinating quorum sensing communication. The production of NAHSLs was investigated in a set of reference strains and recently-collected isolates, which belong to six species and share the ability to infect the potato host plant. All the pathogens produced different NAHSLs, among which the 3-oxo-hexanoyl- and the 3-oxo-octanoyl-l-homoserine lactones represent at least 90% of total produced NAHSL-amounts. The level of NAHSLs varied from 0.6 to 2 pg/cfu. The involvement of NAHSLs in tuber maceration was investigated by electroporating a quorum quenching vector in each of the bacterial pathogen strains. All the NAHSL-lactonase expressing strains produced a lower amount of NAHSLs as compared to those harboring the empty vector. Moreover, all except *Dickeya dadantii* 3937 induced a lower level of symptoms in potato tuber assay. Noticeably, aggressiveness appeared to be independent of both nature and amount of produced signals. This work highlights that quorum sensing similarly contributed to virulence in most of the tested *Pectobacterium* and *Dickeya*, even the strains had been isolated recently or during the past decades. Thus, these key regulatory-molecules appear as credible targets for developing anti-virulence strategies against these plant pathogens.

## 1. Introduction

Several *Proteobacteria* synthesize *N*-acyl homoserine lactones (NAHSLs) that allow synchronizing the expression of several collective behaviors (for a review see [1]), a mechanism called quorum sensing (QS) by Fuqua *et al.* [2]. Among them, numerous plant-pathogenic bacteria produce NAHSLs, which are involved in aggressiveness and colonization of plant-host [3]. The pectinolytic phytopathogens belonging to *Dickeya* and *Pectobacterium* genera, formerly grouped in the same *Erwinia* genus, are responsible for similar pathologies (*i.e*., blackleg, soft-rot) compromising plant survival as well as integrity of harvested vegetal products. In *Pectobacterium* genus, QS belongs to a complex regulatory cascade controlling the production and exportation of carbapenem antibiotic and numerous lytic enzymes [4,5]. In *Dickeya*, the contribution of QS to virulence is less documented and needs to be clarified [6–8].

The involvement of QS in virulence was intensely investigated in *Pectobacterium atrosepticum*, a particular virulent potato pathogen (for review see [9]). Initially, bacteria are present in infection areas such as plant wounds and accumulate a low level of NAHSLs. Then, they multiply actively to reach the quorum cell density and the associated critical concentration of NAHSL, which trigger the production of the macerating enzymes [10–12]. As a result of these observations, NAHSL-based QS system had been consequently proposed as the target for a biocontrol approach using NAHSL-degrading bacterial strains, which aims at reducing the expression of virulence in *Pectobacterium atrosepticum* [13,14].

In addition to the classical *Pectobacterium atrosepticum*, which causes blackleg in temperate regions, several soft-rot *Pectobacterium* and *Dickeya* bacterial species, including numerous recent isolates and the emerging species “*Dickeya solani*”, are able to cause damage to the potato *Solanum tuberosum* L. and other important crops for which protecting methods are lacking [15–22]. The question rose about the relevance of NAHSL-targeting strategies for protecting crops against these pathogens. To address this question, the nature and relative abundance of NAHSL signaling molecules which were synthesized by potato soft-rot bacteria were compared in a set of *Dickeya* and *Pectobacterium* isolates, including recent isolates found in potato fields throughout Western Europe. In a second step, involvement of NAHSLs in tuber soft-rot was evaluated using a quorum quenching approach. This work revealed the key role of NAHSLs and QS-regulation in soft-rot induction by a wide range of pectinolytic bacteria.

## 2. Experimental Section

### 2.1. Bacterial Strains and Plasmids

The characteristics of the bacterial strains and plasmids used in this work are presented in Table 1. Two panels of six strains each were compared for signal production. One is composed of reference strains, the other of soft-rot bacteria recently isolated from potato plants affected by blackleg. Field potato strains were characterized by both biochemical tests [16,23] and molecular methods using specific primers set for *P. atrosepticum*, *Pectobacterium carotovorum* and *Dickeya* spp. [24]. Rep-PCR genomic fingerprinting characterization was performed to identify “*D. solani*” and *D. dianthicola* [19].

The plasmid pME6010 [28] and its derivative expressing the lactonase-encoding gene *attM*, pMIR102 [29], were introduced into *Pectobacterium* and *Dickeya* strains by electroporation.

### 2.2. Growth Media and Conditions

*Pectobacterium* and *Dickeya* strains were cultivated at 25 °C in polygalacturonic acid (PGA) mineral salt medium [25] the composition of which was modified as follows: K_2_HPO_4_, 16.266 g/L; KH_2_PO_4_, 899 mg/L; (NH_4_)_2_SO_4_, 1.2 g/L; MgSO_4_.6H_2_O, 818 mg/L; CaCl_2_, 75 mg/L (pH 8.0, Merck, Fontenay-sous-bois, France) and polygalacturonic acid 4.0 g/L (potassium salt, Sigma-Aldrich, St. Quentin Fallavier, France). Batch cultures were performed under gyratory agitation (180 rpm) in Erlenmeyer flasks in which the liquid medium is 10% of the total flask volume. Batch precultures and cultures were made in the same experimental conditions. Bacterial growth was monitored by measuring optical density (OD) at 580 nm. The initial OD_580_ of the cultures was usually 0.05. For each strain, at least three independent cultures were made. When necessary, growth media were supplemented with tetracycline (10 mg/L, Sigma-Aldrich).

### 2.3. NAHSL Standards, Extraction of Supernatants and NAHSL Assays

NAHSLs were extracted and analyzed as described previously [30,31]. Briefly, the synthetic standards (Sigma-Aldrich) and stock solutions prepared in high performance liquid chromatography (HPLC)-grade ethyl acetate (Fisher Scientific, Courtaboeuf, France) were stored at −20 °C. The supernatants (1 mL) were extracted twice with equal volumes of ethyl acetate. The combined extracts were dried over anhydrous magnesium sulphate (MgSO_4_, Merck), evaporated to dryness, dissolved in 500 μL of HPLC-grade ethyl acetate and stored at −20 °C until analysis.

Concentrated extracts were analyzed by on-line liquid chromatography mass spectrometry (LC-MS-MS). They were applied to a C18 reverse-phase HPLC column (Agilent Hypersyl ODS, 250 4.6 mm, particle size 5 μm, Interchim, Montluçon, France) using an Agilent Technologies Series 1100 vacuum degasser, LC pump and autosampler (Hewlett Packard, Boeblingen, Germany). The elution procedure consisted of an isocratic profile of methanol-water (50:50, v/v) for 10 min at a flow rate of 0.4 mL/min, followed by a linear gradient from 50% to 90% methanol in water over 15 min, and an isocratic profile over 25 min. The post-column flow was split (1/10) by a micro-splitter valve (Upchurch Scientific, Oak Harbor, WA, USA) and a mixture of 5 mM ammonium acetate (Merck) and 0.05% trifluoroacetic acid (Sigma-Aldrich) in methanol-water (50:50, v/v, 150 μL/h) was added using a Cole-Parmer syringe pump. Detection was made by electrospray ionisation-ion trap mass spectrometry using a Bruker Esquire-LC spectrometer (Bruker Daltonic, Wissembourg, France) under positive-ion conditions. The identification of NAHSLs from supernatant extracts was carried out by comparison with synthetic standards, based on three criteria: HPLC retention times, MS-MS fragment ions of the molecular [M+H]^+^ ions (four product ions: the lactone ring *m*/*z* 102, [M+H–101]^+^ ion corresponding to the acyl chain, [M+H–H_2_O]^+^ and [M+H–CO]^+^ ions) and on their relative intensities. The chromatographic peak area of the *m*/*z* 102 ion was measured for quantification [30].

### 2.4. Aggressiveness Test on Potato Tubers

Overnight cultures of bacterial strain harboring pME6010 or pMIR102 were washed twice in 0.8% NaCl. Each tuber of *S. tuberosum* var. Allians was inoculated with 10 μL of the cell suspension, which was adjusted to 1.0 at OD_580nm_. At least 8 tubers were inoculated and then incubated at 24 °C. Five days post-infection, tubers were cut in half and the observed symptoms were categorized into four classes according to diameter (D) of the maceration zone: 1, no maceration; 2, low maceration (D < 2 mm); 3, moderate maceration (2 < D < 5 mm); 4, strong maceration (D > 5 mm). The Mann and Whitney test (α = 0.05) was used to analyze the maceration categories.

## 3. Results and Discussion

Two sets of *Pectobacterium* and *Dickeya* strains (Table 1) were compared for NAHSL production and virulence assays. The first panel was composed of three type strains used as international taxonomic references of *P. atrosepticum*, *Pectobacterium carotovorum* and *Dickeya chrysanthemi* species, isolated in the 1950s. Three other strains which are commonly used for virulence studies were included in this panel: (*i*) the psychrotroph strain *P. atrosepticum* CFBP 6276 known both for its virulence on the potato and for the unusual ability among this species to induce a hypersensitive reaction in non host plant [10,25]; (*ii*) the strain *P. carotovorum* EC153, isolated from rotting bell peppers fruit shipped from Mexico and which NAHSL production and virulence were exceptionally enabled at elevated temperatures above 34 °C [26]; and (*iii*) *Dickeya dadantii* 3937, a mesophile strain isolated from the African violet, widely used as a strain model for research in molecular biology [32], found to be highly virulent on various host plants (V. Hélias, *personnal communication*), and from which the entire genome has recently been sequenced [33].

The second set of strains was composed of six recent isolates, between 1998 to 2008, from potato blackleg wounds. They represent the six soft-rot potato species currently encountered in European soils, including a representative member of the emerging *Dickeya* clade, provisionally called “*D. solani*” [17,21,22].

### 3.1. Characterization of NAHSL Signaling Molecules Produced by Potato Soft-Rot Bacteria

**Figure 1.**
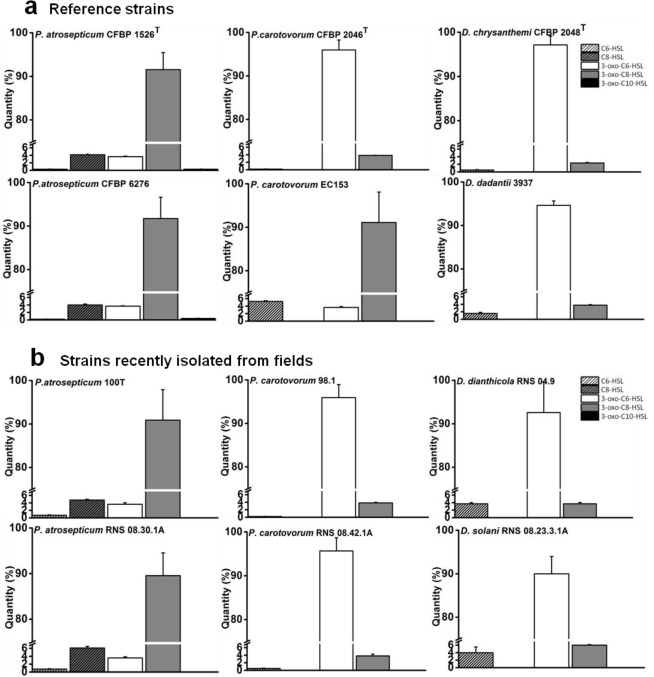
Abundance and diversity of *N*-acyl homoserine Lactone molecules produced by *Pectobacterium* and *Dickeya* strains. NAHSLs were extracted from PGA medium culture supernatants of reference strains (**a**) and recent strains isolated from fields (**b**) during the transition from exponential to stationary phases. On-line liquid chromatography mass spectrometry was used to identify and quantify *N*-hexanoyl-l-homoserine lactone (slashed white bar), *N*-octanoyl-l-homoserine lactone (slashed gray bar), *N*-3-oxo-hexanoyl-l-homoserine lactone (white bar), *N*-3-oxo-l-octanoyl-homoserine lactone (gray bar) and *N*-3-oxo-decanoyl-l-homoserine lactone (black bar). Values are the average of three independent experiments.

**Figure 2.**
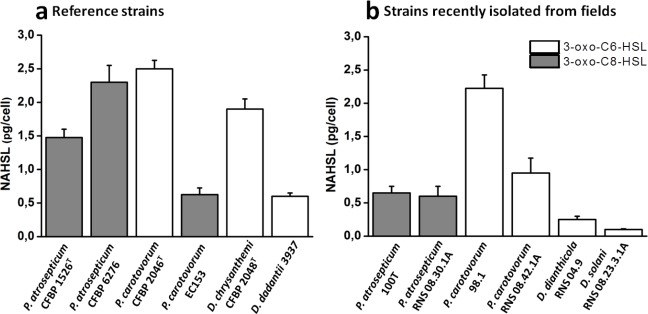
Concentrations of the main *N*-acyl homoserine lactones produced by *Pectobacterium* and *Dickeya* strains. NAHSLs were extracted from PGA medium culture supernatants of reference strains (**a**) and recent strains isolated from fields (**b**) during the transition from exponential to stationary phases and quantified by HPLC coupled to mass spectrometry. For each point, at least 3 independent cultures were analyzed, with standard deviation shown. Legend: white bars, *N-*3-oxo-hexanoyl-l-homoserine lactone; gray bars, *N-*3-oxo-octanoyl-l-homoserine lactone.

The characterization of NAHSL signal molecules was carried out on a mineral salt medium supplemented with PGA, a plant cell-wall compound which contributes to induce the synthesis of signaling molecules and virulence factors as *in situ* conditions [10]. Culture supernatants were recovered during the transition from exponential-phase to stationary-phase growth, when the cell density reached the quorum and the NAHSL concentration is the highest. Produced NAHSLs were identified and quantified using HPLC coupled with mass spectrometry. In these experimental conditions, all *P. atrosepticum* strains produced mainly (90%) *N*-3-oxo-octanoyl-l-HSL (3-oxo-C8-HSL) and a low percent of *N*-octanoyl-l-HSL (C8-HSL) and *N*-3-oxo-hexanoyl-l-HSL (3-oxo-C6-HSL). They also synthesized traces (<1%) of *N*-hexanoyl-l-HSL (C6-HSL) and *N*-3-oxo-decanoyl-l-HSL (3-oxo-C10-HSL) (Figure 1). Among these strains, *P. atrosepticum* CFBP 6276 and 1526^T^ were the largest producers of 3-oxo-C8-HSL with amounts greater than 1 pg/cfu (Figure 2). The other *P. atrosepticum* strains displayed lower production of the major NAHSL with about 0.6 pg/cfu. All *Dickeya* spp. strains and three of the four studied *P. carotovorum* strains produced mainly 3-oxo-C6-HSL and minor percentages of 3-oxo-C8-HSL (Figure 1).

The exception concerns the *P. carotovorum* EC153 strain which demonstrated the same NAHSL production patterns as *P. atrosepticum* strains with the production of about 0.6 pg/cfu of 3-oxo-C8-HSL (Figure 2). The largest producers of 3-oxo-C6-HSL were represented by *P. carotovorum* 2046 and 98.1 strains and *D. chrysanthemi* 2048^T^ strain with amounts greater than 2 pg/cfu (Figure 2). Comparisons between *Dickeya* and *Pectobacterium* genera revealed that the NAHSL production is lower in the former than in the latter.

The characterization of NAHSL molecules produced by the twelve strains belonging to *Pectobacterium* and *Dickeya* genera revealed an apparent intraspecies homogeneity in NAHSL production pattern rather than differences between earlier and recent isolates. This is strengthened by the observation of the same NAHSL production profile of both strains from the current taxonomically described species and emerging isolates. Each NAHSL production pattern contained a major NAHSL, 3-oxo-C8-HSL for *P. atrosepticum* strains and 3-oxo-C6-HSL for *Dickeya* strains, the two molecules identified as true QS signals [29,34,35]. These traits are also observable in the literature for some other soft-rot strains [8,36–38]. There is an exception for *P. atrosepticum* SCRI1043, which is the only *P. atrosepticum* studied strain producing 3-oxo-C6-HSL as a signal instead of 3-oxo-C8-HSL [38,39]. 3-oxo-C6-HSL or 3-oxo-C8-HSL production have always coexisted with minor species of NAHSLs, likely to be less specific products of the NAHSL synthase or catabolites resulting from NAHSL turnover [29,34,35]. Contrary to the nature of NAHSL, the amount of each NAHSL species was variable within *P. atrosepticum* strains and the genus *Dickeya*. The intraspecies homogeneity in NAHSL production pattern was not clearly marked in *P. carotovorum* strains, as already observed by Hasegawa *et al.* [26] and Jafra *et al.* [40]. Although a majority of strains produce 3-oxo-C6-HSL, namely three out of the four studied strains, the *P. carotovorum* EC153 strain used in this study and at least another *P. carotovorum* strain (SCC3193) produce 3-oxo-C8-HSL as signaling molecules [26]. The strong heterogeneity of the *P. carotovorum* species and the diversity of their hosts and environmental niches [41,42] could justify these differences in NAHSL production.

### 3.2. Involvement of NAHSL-Based QS in Potato Soft-Rot Virulence

The contribution of QS using NAHSL in disease establishment and development is now well established for *P. atrosepticum* [9]. Indeed, just an insertional mutation in the gene encoding the NAHSL synthase or only the break down of signaling molecules before their release in the microenvironment are sufficient to remove all symptoms on potato tubers [10,31]. These observations led us to consider NAHSL-QS system as a key target for the development of a novel biocontrol strategy based on the catabolism of signal molecules using natural NAHSL degrading bacteria isolated from the potato rhizosphere [14]. The quorum quenching activity exerted by these bacteria can be attributed to at least three enzymatic activities: a lactonase that opens the γ-butyrolactone-ring of NAHSL [43,44]; an acylase that releases a homoserine lactone and a fatty acid, and an oxido-reductase that reduces the 3-oxo-substitution to a hydroxyl on some NAHSL [44,45].

**Figure 3.**
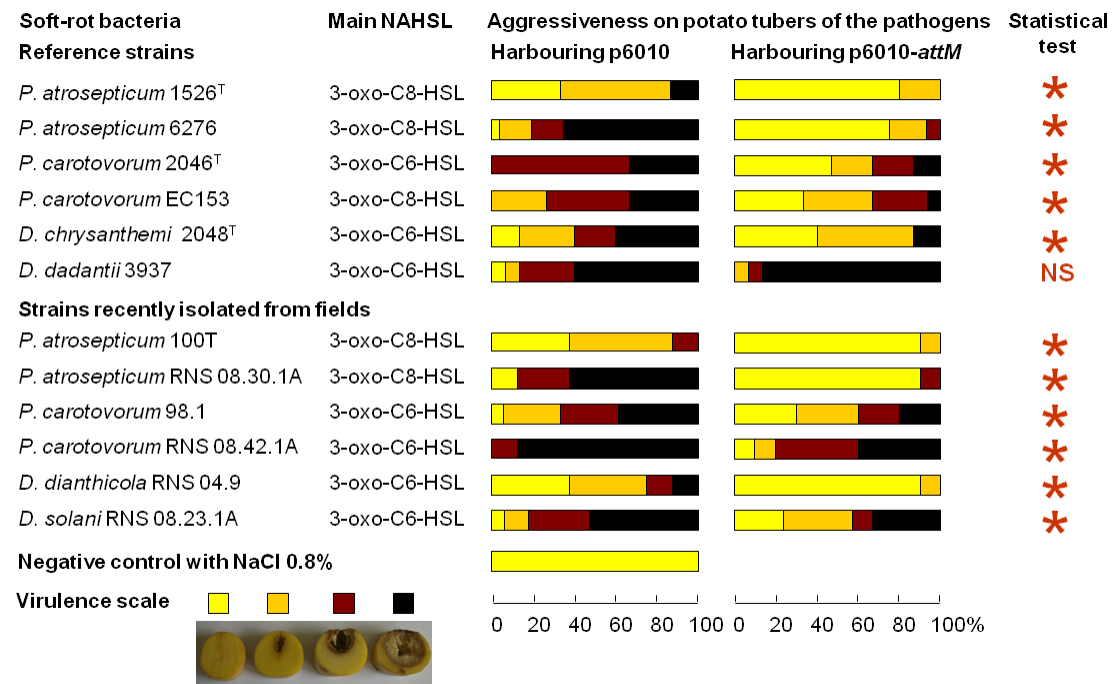
Involvement of NAHSLs in the virulence symptoms on potato tubers. Each tuber of *Solanum tuberosum* var. Allians was inoculated with pathogens harboring the empty vector p6010 or its lactonase AttM-encoding derivative pMIR102. Symptoms were observed five days post-infection and compared using the Mann and Whitney test (α = 0.05). Asterisks indicate statistical differences between symptoms in bacterial derivatives harboring empty and lactonase-expressing vectors. Legend: NS, non-significant.

We used a quorum quenching approach to evaluate the involvement of NAHSLs in virulence of the *Pectobacterium* and *Dickeya* strains. The occlusion of QS signals in the pathogens was based on the introduction of a plasmid carrying a NAHSL-lactonase gene *attM* into each of the twelve studied phytopathogens. The *attM* gene belongs to the *attKLM* operon located on the At plasmid from *Agrobacterium tumefaciens*, which is involved in degradation of γ-butyrolactone, γ-hydroxybutyrate and NAHSLs [29,46,47]. All strains harboring the empty vector p6010 were able to induce tissue maceration five days after potato tuber inoculation (Figure 3). Overall, recent isolates were not more virulent than the reference strains isolated several years before. Symptom severity was variable between *Pectobacterium* and *Dickeya* genera and within *Pectobacterium* species and *Dickeya* genus. For *P. atrosepticum* species, moderate and strong macerations were detected in 10, 80, 10 and 90% of tubers infected by *P. atrosepticum* 1526, 6276, 100T and RNS 08.30.1A, respectively. Interestingly, the two *P. atrosepticum* 100T and RNS 08.30.1A shared the same NAHSL production profile with similar amounts for each NAHSL molecule. Moreover, *P. atrosepticum* RNS 08.30.1A exhibited a similar aggressiveness profile to *D. dadantii* 3937, even if their NAHSL production patterns were different. These observations revealed that symptom severity does not seem to be related to both nature and amount of NAHSL species.

The ectopic expression of the lactonase AttM triggered a decrease about of 100 fold in NAHSL quantity produced by each strain, confirming the capacity of this enzyme in degrading a wide range of NAHSLs (Figure 3). More interestingly, the NAHSL decrease correlated a lower level of symptoms in all pathogen derivatives expressing lactonase AttM as compared those harboring the empty vector. A unique exception was the strain *D. dadantii* 3937 for which no significant symptom attenuation was observed, suggesting no major NAHSL-based QS-dependent expression of the virulence factors [6,48–51].

The AttM expression quenched virulence in the *P. atrosepticum* strains, which produced mainly the 3-oxo-C8-HSL but also in the *P. carotovorum* and *Dickeya* spp. strains, for which the majority produced 3-oxo-C6-HSL as a signal.

This work points out that involvement of NAHSLs in the virulence was conserved in most of the tested *Pectobacterium* and *Dickeya*, which were isolated over the last decades. These key regulatory-molecules appear to be credible targets for developing anti-virulence strategies against the *Pectobacterium* and *Dickeya* plant pathogens. These anti-QS strategies encompass the application of natural or synthetic anti-QS compounds which may target NAHSL-sensors, the construction transgenic plants expressing NAHSL-degrading lactonase, or the application of NAHSL-degrading biocontrol agent, such as the *Rhodococcus erythropolis* (a review by Faure and Dessaux [52]). In the rhizosphere of potato plants, the growth of the NAHSL-degrading *R. erythropolis* may be stimulated by adding γ-caprolactone, a structural analog of NAHSL [13,53].

## 4. Conclusions

By comparing several type strains, well-studied strains from different laboratories [5,36,38,40] and recently isolated strains from fields, this work highlights the key role of QS signals in plant virulence due to soft-rot bacteria. A noticeable exception is the strain *D. dadantii* 3937, in which NAHSLs may not be strongly involved in the expression of virulence factors. These findings reinforce the interest in developing quorum quenching strategies, including biological control based on signal-break down by rhizospheric microbial populations [44,54,55].

## Figures and Tables

**Table 1.** Strains and plasmids used in the present study.

**Strains and plasmids**	**Relevant characteristic(s) or origin (year of plant host isolation)**	**Reference or source**
**Reference strains**		
***Pectobacterium atrosepticum***		
*P. atrosepticum* CFBP 1526^T^	*Solanum tuberosum* (1957)	* CFBP, species type strain
*P. atrosepticum* CFBP 6276	*Solanum tuberosum* (1999)	[25]
***Pectobacterium carotovorum***		
*P. carotovorum* CFBP 2046^T^	*Solanum tuberosum* (1952)	CFBP, species type strain
*P. carotovorum* EC153	*Capsicum annuum* (1951)	[26]
***Dickeya* spp.**		
*D.chrysanthemi* CFBP 2048^T^	*Chrysanthemum morifolium* (1956)	CFBP, type strain
*D. dadantii* 3937	*Saintpaulia ionantha* (1981)	[27]
**Strains recently isolated from fields**		
***Pectobacterium atrosepticum***		
*P. atrosepticum* 100T	*Solanum tuberosum* (2003)	[21]
*P. atrosepticum* RNS 08.30.1A	*Solanum tuberosum* (2008)	V.Hélias/^#^FN3PT’ collection
***Pectobacterium carotovorum***		
*P. carotovorum* 98.1	*Solanum tuberosum* (1998)	[21]
*P. carotovorum* RNS 08.42.1A	*Solanum tuberosum* (2008)	V. Hélias/FN3PT’ collection
***Dickeya* spp.**		
*D. dianthicola* RNS 04.9	*Solanum tuberosum* (2004)	V. Hélias/FN3PT’ collection
“*D. solani*” RNS 08.23.3.1A	*Solanum tuberosum* (2008)	[21]
**Plasmids**		
pME6010	Broad host-range vector, pVS1 derivative, low copy number, Tc^r^	[28]
pMIR102	pME6010 expressing *attM*	[29]

* CFBP: French Collection of Plant associated Bacteria;

# FN3PT: Fédération Nationale des Producteurs de Plants de Pomme de Terre.

## Abbreviations

QS: Quorum sensing
NAHSL: *N*-acyl homoserine lactone
3-oxo-C6-HSL: *N*-3-oxo-hexanoyl-L-HSL
3-oxo-C8-HSL: *N*-3-oxo-octanoyl-L-HSL
HPLC: High performance liquid chromatography
LC-MS: Liquid chromatography mass spectrometry

## References

[1] Ryan R.P., Dow J.M. (2008). Diffusible signals and interspecies communication in bacteria. Microbiology.

[2] Fuqua W.C., Winans S.C., Greenberg E.P. (1994). Quorum sensing in bacteria: The LuxR-LuxI family of cell density-responsive transcriptional regulators. J. Bacteriol.

[3] von Bodman S.B., Bauer W.D., Coplin D.L. (2003). Quorum sensing in plant-pathogenic bacteria. Annu. Rev. Phytopathol.

[4] Barras F., van Gijsegem F., Chatterjee A.K. (1994). Extracellular enzymes and pathogenesis of soft-rot *Erwinia*. Ann. Rev. Phytopathol.

[5] Barnard A.M., Salmond G.P. (2007). Quorum sensing in *Erwinia* species. Anal. Bioanal. Chem.

[6] Nasser W., Bouillant M.L., Salmond G.P.C., Reverchon S. (1998). Characterization of the *Erwinia chrysanthemi expI-expR* locus directing the synthesis of two *N*-acyl-homoserine lactone signal molecules. Mol. Microbiol.

[7] Charkowsky A.O., Gnanamanickam S. (2006). The Soft Rot *Erwinia*. Plant-Associated Bacteria.

[8] Hussain M.B.B., Zhang H.B., Xu J.L., Liu Q., Jiang Z., Zhang L.H. (2008). The acyl-homoserine lactone-type quorum sensing system modulates cell motility and virulence of *Erwinia chrysanthemi* pv. *zeae*. J. Bacteriol.

[9] Liu H., Coulthurst S.J., Pritchard L., Hedley P.E., Ravensdale M., Humphris S., Burr T., Takle G., Brurberg M.B., Birch P.R. (2008). Quorum sensing coordinates brute force and stealth modes of infection in the plant pathogen *Pectobacterium atrosepticum*. PLoS Pathog.

[10] Smadja B., Latour X., Faure D., Chevalier S., Dessaux Y., Orange N. (2004). Involvement of *N*-acylhomoserine lactones throughout the plant infection by *Erwinia carotovora* subsp. *atroseptica* (*Pectobacterium atrosepticum*). Mol. Plant Microbe Interact.

[11] Toth I.K., Newton J.A., Hyman L.J., Lees A.K., Daykin M., Ortori C., Williams P., Fray R.G. (2004). Potato plants genetically modified to produce *N-*acylhomoserine lactones increases susceptibility to soft rot *Erwiniae*. Mol. Plant Microbe Interact.

[12] Maë A., Montesano M., Koiv V., Palva E.T. (2001). Transgenic plants producing the bacterial pheromone *N*-acylhomoserine lactone exhibit enhanced resistance to the bacterial phyto-pathogen *Erwinia carotovora*. Mol. Plant Microbe Interact.

[13] Cirou A., Diallo S., Kurt C., Latour X., Faure D. (2007). Growth promotion of quorum-quenching bacteria in the rhizosphere of *Solanum tuberosum*. Environ. Microbiol.

[14] Crépin A., Barbey C., Cirou A., Tannières M., Orange N., Feuilloley M., Dessaux Y., Burini J.F., Faure D., Latour X. (2011). Biological control of pathogen communication in the rhizosphere: A novel approach applied to potato soft rot due to *Pectobacterium atrosepticum*. Plant Soil.

[15] Ma B., Hibbing M.E., Kim H.S., Reedy R.M., Yedidia I., Breuer J., Glasner D., Perna N.T., Kelman A., Charkowski A.O. (2007). Host range and molecular phylogenies of the soft rot enterobacterial genera *Pectobacterium* and *Dickeya*. Phytopathology.

[16] Laurila J., Ahola V., Lehtinen A., Joutsjoki T., Hannukkala A., Rahkohnen A., Pirhonen M. (2008). Characterization of *Dickeya* strains isolated from potato and river water samples in Finland. Eur. J. Plant Pathol.

[17] Czajkowski R., Grabe G.J., van der Wolf J.M. (2009). Distribution of *Dickeya* spp. and *Pectobacterium carotovorum* subsp. *carotovorum* in naturally infected seed potatoes. Eur. J. Plant Pathol.

[18] Slawiak M., van Beckhoven J.R.C.M., Specksnijder A.G.C.L., Czajkowski R., Grabe G., van der Wolf J.M. (2009). Biochemical and genetical analysis reveal a new clade of biovar 3 *Dickeya* spp. strains isolated from potato in Europe. Eur. J. Plant Pathol.

[19] Tsor (Lahkim) L., Erlich O., Lebiush S., Hazanovsky M., Zig U., Slawiak M., Grabe G., van der Wolf J.M., van de Haar J.J. (2009). Assessment of recent outbreaks of *Dickeya* sp. (syn. *Erwinia chrysanthemi*) slow wilt in potato crops in Israel. Eur. J. Plant Pathol.

[20] Pitman A.R., Harrow S.A., Visnovsky S.B. (2010). Genetic characterization of *Pectobacterium wasabiae* causing soft rot disease of potato in New Zealand. Eur. J. Plant Pathol.

[21] Hélias V., Hamon P., Huchet E., van der Wolf J., Andrivon D. (2011). Two new effective semiselective crystal violet pectate media for isolation of *Pectobacterium* and *Dickeya*. Plant Pathol.

[22] Toth I.K., van der Wolf J.M., Saddler G., Lojkowska E., Hélias V., Pirhonen M., Tsror (Lahkim) L., Elphinstone J.G. (2011). *Dickeya* species: An emerging problem for potato production in Europe. Plant Pathol.

[23] Samson R., Legendre J.B., Christen R., Fisher-Le Saux M., Achouak W., Gardan L. (2005). Transfer of *Pectobacterium chrysanthemi* (Burkholder *et al*. 1953) Brenner *et al*. 1973 and *Brenneria paradisiaca* to the genus *Dickeya* gen. nov. as *Dickeya chrysanthemi* comb. nov. and *Dickeya paradisiaca* comb. nov. and delineation of four novel species: *Dickeya dadantii* sp. nov., *Dickeya dianthicola* sp. nov., *Dickeya dieffenbachiae* sp. nov. and *Dickeya zeae* sp. *nov*. Int. J. Syst. Evol. Microbiol.

[24] Diallo S., Latour X., Groboillot A., Copin P., Smadja B., Orange N., Chevalier S. (2009). Simultaneous and selective detection of two major soft rot pathogens of potato: *Pectobacterium atrosepticum* (*Erwinia carotovora* subsp. *atrosepticum*) and *Dickeya* spp. (*Erwinia chrysanthemi*). Eur. J. Plant Pathol.

[25] Smadja B., Latour X., Trigui S., Burini J.F., Chevalier S., Orange N. (2004). Thermodependence of growth and enzymatic activities implicated in pathogenicity of two *Erwinia carotovora* subspecies (*Pectobacterium* spp.). Can. J. Microbiol.

[26] Hasegawa H., Chatterjee A., Cui Y., Chatterjee A.K. (2005). Elevated temperature enhances virulence of *Erwinia carotovora* subsp. *carotovora* strain EC153 to plants and stimulates production of the quorum-sensing signal, *N*-acyl homoserine lactone, and extracellular proteins. Appl. Environ. Microbiol.

[27] Kotoujansky A., Lemattre M., Boistard P. (1982). Utilization of a thermosensitive episome bearing transposon Tn*10* to isolate Hfr donor strains of *Erwinia carotovora* subsp. *chrysanthemi*. J. Bacteriol.

[28] Heeb S., Itoh Y., Nishijyo T., Schnider U., Keel C., Wade J., Walsh U., O’Gara F., Haas D. (2000). Small, stable shuttle vectors based on the minimal pVS1 replicon for use in gram-negative, plant-associated bacteria. Mol. Plant Microbe Interact.

[29] Carlier A., Uroz S., Smadja B., Latour X., Fray R., Dessaux Y., Faure D. (2003). The Ti plasmid of *Agrobacterium tumefaciens* harbors an *attM*-paralogous gene, *aiiB*, also encoding *N*-acyl homoserine lactonase activity. Appl. Environ. Microbiol.

[30] Morin D., Grasland B., Vallée-Réhel K., Dufau C., Haras D. (2003). On-line high-performance liquid chromatography-mass spectrometric detection and quantification of *N*-acyl homoserine lactones, quorum-sensing signal molecules, in the presence of biological matrices. J. Chromatogr. A.

[31] Latour X., Diallo S., Chevalier S., Morin D., Smadja B., Burini J.F., Haras D., Orange N. (2007). Thermoregulation of *N*-acyl homoserine lactones-based quorum sensing in the soft rot bacterium *Pectobacterium atrosepticum*. Appl. Environ. Microbiol.

[32] Hugouvieux-Cotte-Pattat N., Condemine G., Nasser W., Reverchon S. (1996). Regulation of pectinolysis in *Erwinia chrysanthemi*. Ann. Rev. Microbiol.

[33] Glasner J.D., Yang C.H., Reverchon S., Hugouvieux-Cotte-Pattat N., Condemine G., Bohin J.P., van Gijsegem F., Yang S., Franza T., Expert D. (2011). Genome sequence of the plant-pathogenic bacterium *Dickeya dadantii* 3937. J. Bacteriol.

[34] Brader G., Sjöblom S., Hyytiäinen H., Sims-Huopaniemi K., Palva E.T. (2005). Altering substrate chain length specificity of an acylhomoserine lactone synthase in bacterial communication. J. Biol. Chem.

[35] Welch M., Dutton J.M., Glansdorp F.G., Thomas G.L., Smith D.S., Coulthurst S.J., Barnard A.M.L., Salmond G.P.C., Spring D.R. (2005). Structure-activity relationships of *Erwinia carotovora* quorum sensing signaling molecules. Bioorg. Med. Chem. Lett.

[36] Cha C., Gao P., Chen Y.C., Shaw P., Farrand S.K. (1998). Production of acyl-homoserine lactone quorum-sensing signals by Gram-negative plant-associated bacteria. Mol. Plant Microbe Interact.

[37] Ham J.H., Cui Y., Alfano J.R., Rodriguez-Palenzuela P., Rojas C.M., Chatterjee A.K., Collmer A. (2004). Analysis of *Erwinia chrysanthemi* EC16 *pelE::uidA*, *pel::uidA*, and *hrpN::uidA* mutants reveals strain-specific atypical regulation of the Hrp type III secretion system. Mol. Plant Microbe Interact.

[38] Chatterjee A., Cui Y., Hasegawa H., Leigh N., Dixit V., Chatterjee A.K. (2005). Comparative analysis of two classes of quorum-sensing signaling systems that control production of extracellular proteins and secondary metabolites in *Erwinia carotovora* subspecies. J. Bacteriol.

[39] Toth I.K., Pritchard L., Birch P.R.J. (2006). Comparative genomics reveals what makes an enterobacterial plant pathogen. Annu. Rev. Phytopathol.

[40] Jafra S., Jalink H., van der Schoor R., van der Wolf J.M. (2006). *Pectobacterium carotovorum* subsp. *carotovorum* strains show diversity in production of response to *N*-acyl homoserine lactones. J. Phytopathol.

[41] Gardan L., Gouy C., Christen R., Samson R. (2003). Elevation of three subspecies of *Pectobacterium carotovorum* to species level: *Pectobacterium atrosepticum* sp. nov., *Pectobacterium betavasculorum* sp. nov. and *Pectobacterium wasabiae* sp. *nov*. Int. J. Syst. Evol. Microbiol.

[42] Glasner J.D., Marquez-Villavicencio M., Kim H.S., Jahn C.E., Ma B., Biehl B.S., Rissman A.I., Mole B., Yi X., Yang C.H. (2008). Niche-specificity and the variable fraction of the *Pectobacterium* Pan-genome. Mol. Plant Microbe Interact.

[43] Park S.Y., Hwang B.J., Shin M.H., Kim J.A., Kim H.K., Lee J.K. (2006). *N*-Acylhomoserine lactonase producing *Rhodococcus* spp. with different AHL-degrading activities. FEMS Microbiol. Lett.

[44] Uroz S., Oger P.M., Chapelle E., Adeline M.T., Faure D., Dessaux Y. (2008). A *Rhodococcus* qsdA-encoded enzyme defines a novel class of large-spectrum quorum-quenching lactonases. Appl. Environ. Microbiol.

[45] Uroz S., Chhabra S.R., Camara M., Williams P., Oger P., Dessaux Y. (2005). *N*-Acylhomoserine lactone quorum-sensing molecules are modified and degraded by *Rhodococcus erythropolis* W2 by both amidolytic and novel oxidoreductase activities. Microbiology.

[46] Zhang H.B., Wang L.H., Zhang L.H. (2002). Genetic control of quorum sensing signal turnover in *Agrobacterium tumefaciens*. Proc. Natl. Acad. Sci. USA.

[47] Carlier A., Chevrot R., Dessaux Y., Faure D. (2004). In *Agrobacterium tumefaciens* strain C58, the assimilation of gamma-butyrolactone interferes with the accumulation of the *N*-acyl-homoserine lactone signal. Mol. Plant Microbe Interact.

[48] Yang S., Zhang Q., Guo J., Charkowski A.O., Glick B.R., Ibekwe A.M., Cooksey D.A., Yang C.H. (2007). Global effect of indole-3-acetic acid biosynthesis on multiple virulence factors of *Erwinia chrysanthemi* 3937. Appl. Environ. Microbiol.

[49] Yang S., Peng Q., San Francisco M., Wang Y., Zeng Q., Yang C.H. (2008). Type III secretion system genes of *Dickeya dadantii* 3937 are induced by plant phenolic acids. PLoS One.

[50] Effantin G., Rivasseau C., Gromova M., Bligny R., Hugouvieux-Cotte-Pattat N. (2011). Massive production of butanediol during plant infection by phytopathogenic bacteria of the genera *Dickeya* and *Pectobacterium*. Mol. Microbiol.

[51] Mhedbi-Hajri N., Malfatti P., Pédron J., Gaubert S., Reverchon S., van Gijsegem F. (2011). PecS is an important player in the regulatory network governing the coordinated expression of virulence genes during the interaction between *Dickeya dadantii* 3937 and plants. Environ. Microbiol.

[52] Faure D., Dessaux Y. (2007). Quorum sensing as a target for developing control strategies for the plant pathogen *Pectobacterium*. Eur. J. Plant Pathol.

[53] Cirou A., Raffoux A., Diallo S., Latour X., Dessaux Y., Faure D. (2011). Gamma-caprolactone stimulates the growth of quorum-quenching *Rhodococcus* populations in a large-scale hydroponic system for culturing *Solanum tuberosum*. Res. Microbiol.

[54] Cirou A., Mondy S., An S., Charrier A., Sarrazin A., Thoison O., DuBow M., Faure D. (2012). Efficient biostimulation of the native and introduced quorum-quenching *Rhodococcus erythropolis* is revealed by a combination of analytical chemistry, microbiology and pyrosequencing. Appl. Environ. Microbiol.

[55] Barbey C., Crépin A., Cirou A., Budin-Verneuil A., Orange N., Feuilloley M., Faure D., Dessaux Y., Burini J.F., Latour X. (2012). Catabolic pathway of gamma-caprolactone in the biocontrol agent *Rhodococcus erythropolis*. J. Proteome Res.

